# Occupational Asthma: New Low-Molecular-Weight Causal Agents, 2000–2010

**DOI:** 10.1155/2012/597306

**Published:** 2012-04-04

**Authors:** J. A. Pralong, A. Cartier, O. Vandenplas, M. Labrecque

**Affiliations:** ^1^Department of Chest Medicine, Sacré-Cœur Hospital, University of Montreal, QC, Canada H4J 1C5; ^2^Department of Chest Medicine, Mont-Godinne Hospital, Université Catholique de Louvain, 5530 Yvoir, Belgium

## Abstract

*Background*. More than 400 agents have been documented as causing occupational asthma (OA). The list of low-molecular-weight (LMW) agents that have been identified as potential causes of OA is constantly expanding, emphasizing the need to continually update our knowledge by reviewing the literature. *Objective*. The objective of this paper was to identify all new LMW agents causing occupational asthma reported during the period 2000–2010. *Methods*. A Medline search was performed using the keywords *occupational asthma*, *new allergens*, *new causes*, *and low-molecular-weight agents*. *Results*. We found 39 publications describing 41 new LMW causal agents, which belonged to the following categories: drugs (*n* = 12), wood dust (*n* = 11), chemicals (*n* = 8), metals (*n* = 4), biocides (*n* = 3), and miscellaneous (*n* = 3). The diagnosis of OA was confirmed through SIC for 35 of 41 agents, peak expiratory flow monitoring for three (3) agents, and the clinical history alone for three (3) agents. Immunological tests provided evidence supporting an IgE-mediated mechanism for eight (8) (20%) of the newly described agents. *Conclusion*. This paper highlights the importance of being alert to the occurrence of new LMW sensitizers, which can elicit OA. The immunological mechanism is explained by a type I hypersensitivity reaction in 20% of all newly described LMW agents.

## 1. Introduction

 Occupational asthma (OA) is defined as “a disease characterized by variable airflow limitation and/or hyper-responsiveness and/or inflammation due to causes and conditions attributable to a particular occupational environment and not to stimuli encountered outside the workplace” [1]. Two types of OA are distinguished based on their appearance after a latency period or in absence of a latency period. The most frequent type, which is usually quoted as “occupational asthma,” appears after a latency period eventually leading to sensitization (either allergic or through unknown immunological mechanisms). The other category does not require a latency period and includes irritant-induced asthma or reactive airway dysfunction syndrome (RADS), which may occur after single or multiple exposures to high concentrations of nonspecific irritants [2]. 

The diagnosis of OA is often a challenge. A stepwise approach is required to confirm the diagnosis as recently reviewed in a consensus statement of the American College of Chest Physicians on diagnosis and management of work-related asthma [3]. This includes a thorough questionnaire on symptoms and work description, with objective confirmation of the diagnosis of asthma, either by confirming reversible airflow obstruction or by documenting increased nonallergic bronchial responsiveness (although the latter may be absent if away from work). Immunological testings (such as skin prick tests, documentation of specific IgE or IgG) are useful to document sensitization but do not confirm the diagnosis of OA. Assessment of the relationship of asthma to work is done by monitoring of peak expiratory flows, methacholine or histamine inhalation challenges, sputum induction at and off work, and/or by specific inhalation challenges (SIC), which are considered the reference standard where available. The description of these methods is beyond the scope of this paper and the reader can refer to the book *Asthma in the workplace* published by Bernstein et al. [4] for more details.

 Asthma is one of the most prevalent respiratory diseases in occupational settings. The prevalence of OA is estimated between 10 and 16% of all new adult-onset asthma [3, 5–7]. Its incidence is estimated between 22 and 40 new cases per million of active workers each year [8]. The financial burden of OA is very high. The total lifetime cost for all new cases of OA diagnosed in 2003 in the UK was estimated to be between *£*70–100 million [9].

 More than 400 distinct agents have been documented as causing OA [10], and their number is steadily growing with the development of industrial processes. However, knowing which agents are potential airway sensitizers is an important step for early identification of OA among asthmatic patients in order to adequately manage them and at the same time, to prevent new cases from occurring. Classically, the agents responsible for OA are divided according to their molecular weight. High-molecular-weight (HMW) agents (>10 kDa [11]) include animal and vegetal origin proteins and microorganisms. Low-molecular-weight (LMW) agents are represented by wood dust, drugs, metals, and chemicals. The mechanisms leading to immunological sensitization to low molecular weight agents remain largely uncertain [12].

 The objective of the present study was to review all new LMW agents causing occupational asthma with a latency period reported between 2000 and 2010. This paper focused on LMW agents given that two recent reviews had already addressed the major HMW agents seen in the food and seafood industry [13, 14], and that the most recent reviews of LMW agents dated back to 2000 and 2001 [15, 16].

## 2. Methods

 We searched Medline for publications or abstracts in English using the keywords *occupational asthma, new allergens, new causes, *and *low-molecular-weight agents* between 2000 and 2010.

## 3. Results

 The bibliographic search identified 39 case reports describing 41 new LMW agents causing OA during the period 2000–2010. These agents are listed in Table 1. Among these 41 new LMW agents recognized as causing OA, twelve (12) belonged to the *Drugs* category, eleven (11) to the *Wood Dust* category, four (4) to the *Metals* category, and eight (8) to the *Chemicals* category. We also found three (3) biocides, two (2) fungicides, and one (1) anhydride salt classified as miscellaneous.

Most of the identified articles are case reports or short series, including a total of 62 subjects. Among these, 33 (53%) experienced concomitant rhinitis and four (4) (6%) suffered from dermatitis (urticaria or contact dermatitis).

 As shown in Table 1, the diagnosis workup included monitoring of peak expiratory flow (PEF) at and off work in 29 (47%) cases, evaluation of nonspecific bronchial responsiveness (NSBR) in 52 (84%) cases, and determination of sensitization by skin prick tests (SPTs) in 26 (42%) cases or specific immunoglobulin E (IgE) in 22 (35%) cases and specific inhalation challenges (SIC) in 48 (77%) cases.

### 3.1. Drugs

Between 2000 and 2010, twelve (12) new drugs were found to induce OA [17–28] (Table 1), including six (6) antibiotic components: 7-aminocephalosporanic acid (7-ACSA), 7-amino-3-thiomethyl-3-cephalosporanic acid (7-TACA), cefteram, vancomycin, colistin, and thiamphenicol. Three (15%) of these cases occurred in health care workers and 15 (75%) in pharmaceutical employees.

 The diagnosis was confirmed by a positive SIC in 18 of the 19 tested subjects with early (*n* = 10), late (*n* = 6), and atypical (*n* = 1) reactions and was not specified in the remaining case. The SIC was negative in one worker exposed to sevoflurane and equivocal in the same worker exposed to isoflurane. Sixteen (16) workers showed increased NSBR and five (5) had significant PEF variability at and off work. As confirmed by positive SPT and specific IgE, a type I hypersensitivity reaction explained OA in workers exposed to thiamphenicol, 7-ACSA, and cefteram.

 In the case exposed to vancomycin powder [17], specific SPT and IgE were negative, but positive intradermal reaction and histamine release test suggested a direct histamine releasing effect of vancomycin instead of an IgE-mediated mechanism.

 While colistin is known to induce severe bronchospasm by an unknown mechanism, especially in patients with cystic fibrosis [56], Gómez-Ollés et al. reported a case of OA and rhinitis due to inhalation of colistin in which the subject presented an immediate asthmatic reaction during the SIC [18], the mechanism remaining unknown since the determination of specific IgE was negative.

 Park et al. described OA in two (2) workers exposed to 7-ACSA powder (an intermediate metabolite of the synthesis of ceftriaxone) [19]. Both workers developed an early asthmatic reaction when exposed to 7-ACSA but not when exposed to ceftriaxone. Only one clearly had a type I allergic reaction including a positive specific SPT, positive specific IgE antibodies, and an immediate reaction during the SIC, whereas the other had negative specific SPT and IgE.

 Three (3) cases of OA in pharmaceutical workers exposed to thiamphenicol (a derivative of chloramphenicol) were reported, two (2) of them having presented a type I hypersensitivity reaction (positive specific SPT, positive specific IgE antibodies, and an early reaction during the SIC) and the third one, probably a non-IgE-mediated reaction (negative specific SPT, specific IgE antibodies, and a late asthmatic reaction) [20]. Other categories of drugs were identified as being able to induce OA (Table 1).

### 3.2. Wood Dust

 Eleven (11) new wood species have been associated with OA [29–38] (Table 1), the majority being exotic species originating from Africa, South America, or Asia. The cases were reported in carpenters and wood workers. Eight (8) of the twelve (12) reported patients (67%) also had rhinitis and one (1) case had dermatitis. In all but one of the subjects, the diagnoses had been confirmed by a positive SIC with early (*n* = 5), late (*n* = 3) and dual (*n* = 3) reactions. The diagnosis of the remaining case (exposed to sapele) was mainly based on history. Noticeably, the worker exposed to cedroarana had a negative methacholine test both before and after the SIC, despite an early asthmatic reaction [32]. Induced sputum analysis performed after the SIC showed an increased eosinophil count in all of the four (4) patients on whom this technique had been performed. Specific SPTs were positive in four (4) out of nine (9) patients. Specific IgE antibodies were positive in four (4) of the eight (8) patients tested, confirming a type I allergic reaction for four (4), distinct wood species (cedroarana, angelim pedra, antiaris and sapele).

### 3.3. Metals

 Four (4) metals have been reported as causal agents of OA [39–42] (Table 1). Most of the metals causing OA belong to the transitional metal series. In the first series of transitional metals, chromium, cobalt, and nickel are all known to induce OA [57–62]. Belonging to the same series, manganese was shown to induce OA in a welder working in a train factory [39].

 Among the second series of transitional metals, palladium is known to cause OA [63], while Merget et al. reported the first case of OA and rhinitis induced by rhodium salts [40] and confirmed by an immediate asthmatic reaction during the SIC. The positive SPT is compatible with a type I hypersensitivity reaction although no specific IgE were detected. Previous studies have shown cross-reaction between platinum salts and rhodium; even though SPT and SIC were positive for both platinum and rhodium in this case, the authors concluded the absence of cross-reactivity.

 Hannu et al. reported another case of OA in a welder exposed to fumes from stellite [41], which is an alloy made of cobalt (60%), chromium (30%), tungsten, and carbon. A positive SIC confirmed the diagnosis when the worker was exposed to stellite welding fumes but not when he was exposed to cobalt or chromium solutions alone.

 Mu$\tilde{\text{n}}$oz et al. reported three (3) cases of OA induced by iron metal welding [42]. The diagnoses were confirmed by SIC, eliciting in two out of three (2/3) workers an atypical response with a rapid and persistent decline in lung function over time and in the last one, a dual response. The analysis of induced sputum revealed an increase in the neutrophil count. Air analysis during the SIC found a high number of metals and gases, none of these components (such as O_3_ or NO_2_) exceeding the threshold limit values. Therefore, it is difficult to know which agent was the direct cause of OA in this precise situation.

### 3.4. Chemicals

 Among chemicals, eight (8) new agents inducing OA have been reported for the period [43–50] (Table 1). Among these, Vandenplas et al. documented a case of OA and rhinitis due to uronium salt, a compound used as a peptide coupling agent [43], ascertained by an early asthmatic reaction during the SIC and positive SPT. They also reported three (3) other workers with skin symptoms and rhinitis, demonstrating positive SPT.

 Occupational asthma was also documented with new fluxes replacing colophony in electronic settings, adipic acid, and dodecanedioic acid gel [44, 45].

 Hnizdo et al. reported an outbreak of OA in a chemical plant, where six (6) cases of OA and rhinitis due to 3-amino-5-mercapto-1,2,4-triazole, a chemical agent used in the production of herbicides, were described [46]. The diagnoses were based on history, increased NSBR, and monitoring of PEF variability at and off work.

### 3.5. Biocides

 Cases of OA to three (3) new biocides have been reported [51–53] (Table 1) in healthcare workers.

 Chlorine releasing agents are often used to disinfect water in swimming pools. The role of these biocides is questioned in the pathogenesis of asthma in swimmers (both recreational and competitive) and children [64–66]. Thickett et al. published the case of two (2) lifeguards and a swimming pool instructor working in three distinct indoor swimming pools and diagnosed with OA [51]. Two (2) of them had significant PEF variability at work, and the diagnosis was confirmed in all of them by either SIC to nitrogen trichloride (a compound of the chloramine family which is found in swimming pool air) in two workers or by a positive poolside challenge test. Two (2) of the workers had normal NSBR when tested. A controlled case sensitized to formaldehyde showed an early reaction when exposed to nitrogen trichloride but not when exposed to distilled water, suggesting that nitrogen trichloride could also be an irritant.

### 3.6. Miscellaneous

 Draper et al. reported two (2) cases of OA in subjects exposed to the new fungicides fluazinam and chlorothalonil [54] which induced late asthmatic reactions during the SIC.

 Keskinen et al. reported a case of OA due to chlorendic anhydride (found in polyester paints) in a mechanic exposed to welding fumes [55]. Chlorendic anhydride belongs to the anhydride group, a group containing several compounds, which can cause OA.

## 4. Discussion

Forty-one (41) new LMW agents have been identified as causes of OA for the period 2000–2010, which represents a mean of four (4) new agents per year.

Ascertaining the presence of asthma through variable airway obstruction and/or increased NSBR is usually considered the first step of the diagnostic approach [3]. Such a test is preferably conducted shortly after a period of work exposure. Because of its high sensitivity, a negative test helps in ruling out the diagnosis of asthma in a symptomatic patient. Among the cases reported during the studied period, increased NSBR was documented in forty-five (45) of the fifty-two (52) subjects (87%) who completed a methacholine or histamine challenge. The NSBR test has been evaluated only before SIC in thirty-two (32) subjects and was increased in twenty-nine (29). In three (3) workers (exposed to colistin, lasamide, and nitrogen trichloride), NSBR was normal before SIC, but the test had not been repeated after the positive challenge. For the remaining twenty (20) workers, the NSBR evaluation had been performed before and after SIC. Twelve (12) subjects had an increased NSBR before SIC, which further increased after the challenge. Four (4) subjects had a normal NSBR before SIC and showed an increased NSBR after SIC and in the remaining four (4), the challenge failed to demonstrate NSBR both before and after SIC (in workers exposed to cedroarana, 7-TACA, nitrogen trichloride, and chlorothalonil). NSBR has only been evaluated at and off work in three (3) subjects exposed to iron fumes. Of the eleven (11) workers with normal baseline NSBR, seven (7) were still being exposed at work at the time of the assessment. The remaining four (4) had already been moved to another workplace, which may explain the negative result of the challenge. Among the eleven (11) workers without baseline NSBR, three (3) (exposed to 5-ASA, uronium salt and eugenol) showed increased sputum eosinophils, suggesting OA or occupational bronchitis.

Therefore, even in the absence of increased NSBR in OA, there is a necessity to continue the diagnostic workup when the history is highly suggestive of OA, especially when the worker has been removed from his job.

Among the reviewed case reports, the diagnosis of OA was confirmed objectively with SIC, which is considered the reference standard in the diagnosis of sensitizer-induced OA [3, 67] for thirty-five (35) of the forty-one (41) described agents. Different types of asthmatic reactions have been described in workers exposed to LMW, isolated late (23%) or atypical (3%) reactions being more frequent when compared to HMW agents (9% and 0%, resp.) [10, 68]. In this paper, the SIC demonstrated an early asthmatic reaction in twenty-two (22) workers (47%), an isolated late reaction in fourteen (14) workers (30%), a dual reaction (an early followed by a late reaction) in seven (7) workers (15%), and an atypical reaction in only three (3) workers (6%). For three (3) agents (vancomycin, 3-amino-5-mercapto-1,2,4-triazole and chlorendic anhydride), the diagnosis was confirmed by PEF monitoring at and off work. Overall, PEF monitoring was positive in 24/29 subjects. Serial peak expiratory flow monitoring is a simple and inexpensive way to evaluate work-related asthma with a sensitivity of 78% and a specificity of 92% when using a computer-based pattern recognition system like Oasys [69]. However, it has the disadvantage of being effort dependent, requiring careful supervision and collaboration of the worker. In the three (3) remaining agents (sapele, artificial flavour, and orthophthalaldehyde), the diagnosis was based solely on the clinical history and improvement of symptoms away from work, without any objective evidence of work-related asthma.

 It is well established that the pathophysiology of OA due to HMW allergens is similar to that of non-OA asthma, involving a type I hypersensitivity reaction mediated by specific IgE antibodies [11, 12]. Eosinophils are the key inflammatory cells found in airway inflammation due to HMW sensitizers. The clinical tests usually find specific IgE antibodies to the HMW agent, specific SPTs are positive, and an increase in eosinophil count is often found in induced sputum analysis after challenge exposure. The presence of specific IgE antibodies has been demonstrated in OA due to some LMW agents, for example, platinum salts or acid anhydrides. However, for the vast majority of LMW allergens, the immunological mechanism remains poorly understood [70]. These LMW compounds act as haptens that combine with self-proteins, creating new antigens recognized as nonself by the immune system, and generating a specific immune response [71]. In this paper, the presence of specific IgE antibodies was documented for eight (8) (20%) of the newly described agents, including thiamphenicol, cefteram, 7-ACSA, cedroarana, angelim pedra, sapele, antiaris, and chlorendic anhydride. Other postulated mechanisms involved are a direct histamine releasing effect for vancomycin-induced OA [17] or an IgG-mediated reaction for falcate-induced OA [31].

 Induced sputum analysis is a validated technique used to assess airway inflammation in OA [72]. In OA due to LMW agents, both eosinophils and neutrophils can be elevated, together or separately. In the present paper, induced sputum was assessed during SIC in twelve (12) patients, and a postchallenge increase in eosinophil counts was documented for eight (8) agents (5-ASA, tali, jatoba, bethabara, manganese, uronium salts, eugenol, and peracetic acid-hydrogen peroxide mixture), whereas an increase in neutrophil count was recorded only for iron. Sputum samples were collected 24 h after SIC, except for bethabara and manganese sampling, which took place the same day as SIC. The measurement of exhaled nitric oxide (eNO) level has been proposed as an alternative method for quantifying airway inflammation, but its role in the occupational setting is not well defined [11, 71]. Only two (2) workers exposed to 5-ASA and dodecanedioic acid, respectively, had such a measurement; it showed an increase in eNO 24 hours after SIC from 32 to 53 ppb, associated with sputum eosinophilia in the worker exposed to 5-ASA and a low result (14.3 ppb) before SIC in the worker exposed to dodecanedioic acid.

 In terms of symptoms, rhinitis is more frequently associated with OA due to HMW allergens (92% of workers with OA compared to 55% of workers exposed to LMW agents) [10, 73]. Therefore, it is not surprising to note that 53% of the workers identified in this paper complained of rhinitis symptoms. It is now believed that occupational asthma and occupational rhinitis (OR) are part of the same disease process [73, 74]. Moreover, OR can be considered a risk factor in the development of OA, even if the proportion of workers with OR eventually developing OA remains uncertain [73].

 According to recent reviews, the prevalence of OA tends to be stable over time [75] or decrease in the last years [76, 77] in industrialized countries, although this may be due to underreporting [78]. Despite this possible reduction in the prevalence of OA in certain industries, new sensitizers are still reported each year. Therefore, vigilance is still required.

Predicting the risk of a new chemical agent as being a potential respiratory sensitizer could be a useful tool in the primary prevention of OA. The (quantitative) structure-activity relationship model ((Q)SARs) has been developed by the drug industry to evaluate the potential toxic effect of drugs in different situations. The method is based on the link between the chemical structure and the health effects. Agius et al. developed and validated the QSAR model to predict the asthmagenic potential of LMW organic agents [79]. They showed that some chemical functional groups (i.e., nitrogen and oxygen containing groups such as isocyanate, amine, acid anhydride, and carbonyl) were associated with OA hazard, especially when these groups were present more than once in the same molecule [80]. Their model was able to correctly identify 90% of LMW organic agents as asthmagenic or not. Using a threshold hazard index of 0.5, the sensitivity and specificity of the model in the external validation group were 86% and 99% respectively, while the negative predictive value was 100%. The model can be accessed through the following web page: http://www.coeh.man.ac.uk/research/asthma/. A hazard index has been calculated for fifteen (15) LMW out of the forty-one (41) reported during the period 2000–2010 [80, 81]. The hazard index was surprisingly low for three (3) agents (fluazinam, sevoflurane, and eugenol), but for the twelve (12) others, it ranged between 0.71 and 1, confirming the sensitizing hazard of these agents.

## 5. Conclusion

The list of LMW agents responsible for OA is continuously growing as shown in this paper with forty-one (41) new agents being reported during the period 2000–2010. The involved immunological mechanisms are various and now more often than before include IgE-mediated process in the newly reported agents (20%).

Physicians should be cautious when diagnosing in that they should not rely solely on lists of agents known to be sensitizers when considering further investigation of workers with work-related symptoms of asthma.

## Figures and Tables

**Table 1:** New agents causing OA.

Substances	Ref- erence	Workplace	No. of cases	Symptoms	Immunological tests		Functional tests			SIC	Remark
					SPT	IgE	Increased NSBR	PEF			
Drugs											

Vancomycin	[17]	Pharmaceutical company	1	A, R	−	−	+	+	ND	NA	Intradermal test +/Histamine release test +
Colistin	[18]	Pharmaceutical company	1	A, R	ND	−	−	ND	+	EAR	NSBR negative before SIC
7-ACSA	[19]	Pharmaceutical company	2	A (2/2), R (1/2)	+ (1/2)	+ (1/2)	+ (2/2)	ND	+ (2/2)	EAR (1/2) NS (1/2)	
Thiamphenicol	[20]	Pharmaceutical company	3	A (3/3), R (3/3)	+ (2/3)	+ (2/3)	+ (3/3)	ND	+ (3/3)	EAR (2/3) LAR(1/3)	
Cefteram	[21]	Pharmaceutical company	2	A	+ (2/2)	+ (2/2)	+ (2/2)	ND	+ (2/2)	EAR (2/2)	
7-TACA	[22]	Pharmaceutical company	1	A, R	ND	ND	−	ND	+	EAR	Increased nasal eosino count after SIC/NSBR negative before and after SIC
Thiamine	[23]	Cereal manufacture	2	A (2/2)	ND	− (1/1)	+ (1/1)	+ (2/2)	+ (2/2)	LAR (2/2)	Hypoxemia after SIC: pO2 45 mm Hg
Lasamide and precursors	[24]	Pharmaceutical company	3	A (3/3), R (3/3)	ND	ND	+ (2/3)	ND	+ (3/3)	EAR (3/3)	NSBR negative before SIC in 1 subject
Aescin	[25]	Pharmaceutical company	1	A, R	ND	ND	+	+	+	AAR	
Sevoflurane and isoflurane	[26]	Hospital, anaesthetic staff	2	A (2/2), R (1/2), AR (1/2)	ND	ND	+ (2/2)	+ (1/2)	+ (1/2)	LAR	
Mitoxantrone	[27]	Hospital, oncology staff	1	A, R	ND	ND	+	ND	+	LAR	BAL: increase in neutrophils, lymphocytes and eosino after SIC
5-ASA	[28]	Pharmaceutical company	1	A	−	ND	+	ND	+	LAR	Increase in sputum eosino after SIC/Increase in eNO level after SIC

Total: 12			20	A 20/R 13/D 0	5/9	5/10	16/19	5/6	18/19	EAR 10/LAR 6/AAR 1/NS 1	

Wood dust											

Tali	[29]	Carpentry	2	A (2/2), R (2/2)	− (0/2)	− (0/2)	+ (2/2)	+ (2/2)	+ (2/2)	LAR (1/2) DAR (1/2)	Increase in sputum eosino after SIC (2/2)
Jatoba	[29]	Carpentry	1	A, R	−	−	+	+	+	EAR	Increase in sputum eosino after SIC
Chengal	[30]	Carpentry	1	A, R	ND	ND	ND	+	+	EAR	
Falcata	[31]	Wood furniture plant	1	A	ND	ND	ND	ND	+	EAR	Intradermal test +/Specific IgG +
Cedroarana	[32]	Carpentry	1	A, R	+	+	−	ND	+	EAR	Nasal provocation test +/NSBR negative before and after SIC
Bethabara	[33]	Railway platform	1	A	ND	ND	+	ND	+	LAR	Increase in sputum eosino after SIC
Angelim pedra	[34]	Carpentry	1	A, R	+	+	+	ND	+	EAR	
Ipe	[35]	Wood work	1	A	+	−	ND	ND	+	LAR	
Antiaris	[36]	Door manufacture	1	A, R	+	+	+	ND	+	DAR	Conjunctival provocation test +
African cherry	[37]	Carpentry	1	A	−	ND	+	ND	+	DAR	
Sapele	[38]	Carpentry	1	A, R, D	−	+	ND	ND	ND	NA	

Total: 11			12	A 12/R 8/D1	4/9	4/8	7/8	4/4	11/11	EAR 5/LAR 3/DAR 3	

Metals											

Manganese	[39]	Welding	1	A	−	ND	+	+	+	EAR	Increase in sputum eosino and basophils after SIC
Rhodium	[40]	Electroplating plant	1	A, R	+	−	+	ND	+	EAR	
Stellite	[41]	Machine manufacture	1	A	−	ND	+	+	+	DAR	
Iron (fumes)	[42]	Welding	3	A (3/3)	ND	ND	+ (3/3)	+ (1/3)	+ (3/3)	DAR (1/3) AAR (2/3)	Increase in sputum neutrophils after SIC (3/3)

Total: 4			6	A 6/R 1/D 0	1/3	0/1	6/6	3/5	6/6	EAR 2/DAR 2/AAR 2	

Chemicals											

Uronium salts	[43]	Peptides synthesis lab	1	A, R	+	−	+	ND	+	EAR	Increase in sputum eosino after SIC
Dodecanedioic acid gel flux	[44]	Electronics company	1	A, R	ND	ND	+	+	+	EAR	Low eNO level before SIC
Adipic acid flux	[45]	Soldering	1	A	ND	ND	+	+	+	LAR	
3-amino-5-mercapto-1,2,4-triazole	[46]	Production of herbicides	6	A (6/6), R (6/6)	ND	ND	+ (6/6)	+ (3/4)	ND	NA	
Sodium disulphite	[47]	Lobster fishing	1	A	−	−	+	+	+	EAR	
Tetramethrin	[48]	Insect extermination firm	1	A	−	ND	+	ND	+	DAR	
Eugenol	[49]	Hairdressing salon	1	A, R, D	−	ND	+	ND	+	LAR	Increase in sputum eosino and lymphocytes after SIC
Artificial flavour	[50]	Popcorn popping company	3	A	ND	ND	ND	ND	ND	NA	

Total: 8			15	A 15/R 9/D1	1/4	0/2	12/12	6/7	6/6	EAR 3/LAR 2/DAR 1	

Biocides											

Nitrogen trichloride	[51]	Swimming-pool	3	A	ND	ND	+ (1/3)	+ (2/3)	+ (3/3)	EAR (2/3) DAR (1/3)	NSBR negative before SIC in 1 subject and negative before and after SIC in another subject
PA-HP	[52]	Endoscopic unit	2	A (2/2), R (2/2)	ND	ND	+ (2/2)	+ (1/1)	+ (1/1)	LAR	Increase in sputum eosino after SIC (1/1)
Ortho-phthalaldehyde	[53]	Endoscopic unit	1	A, D	ND	ND	ND	ND	ND	NA	

Total: 3			6	A 6/R 2/D1	ND	ND	3/5	3/4	4/4	EAR 2/LAR 1/DAR 1	

Miscellaneous											

Fluazinam	[54]	Fungicides manufacture	1	A	ND	ND	+	+	+	LAR	
Chlorothalonil	[54]	Fungicides manufacture	1	A	ND	ND	−	+	+	LAR	NSBR negative before SIC and borderline after SIC
Chlorendic anhydride	[55]	Mechanic work	1	A, D	+	+	ND	+	ND	NA	

Total: 3			3	A 3/R 0/D1	1/1	1/1	1/2	3/3	2/2	LAR 2	

Total: 41			62	A 62/R 33/D 4	12/26	10/22	45/52	24/29	47/48	EAR 22/LAR 14/DAR 7/AAR 3/NS 1/47	

Legend: A: asthma, R: rhinitis, D: dermatitis; U: urticaria; AR: anaphylactic reaction; PEF: peak expiratory flow, SIC: specific inhalation challenge SPT: skin prick test, IgE: specific immunoglobulin E, NSBR: nonspecific bronchial responsiveness EARs: early asthmatic reaction, LAR: late asthmatic reaction, DAR: dual asthmatic reaction, AAR: atypical asthmatic reaction ND: not done, NA: not available, NS: not specified, eosino: eosinophils, 7-ACSA: 7-aminocephalosporanic acid, 7-TACA: 7-amino-3-thiomethyl-3-cephalosporanic acid, 5-ASA: 5-aminosalicylic acid, PA-PH: peracetic acid-hydrogen peroxyde mixture, BAL: bronchoalveolar lavage, eNO: exhaled nitric oxyde, IgG: immunoglobulin G.
